# Advances in Antibody Preparation Techniques for Immunoassays of Total Aflatoxin in Food

**DOI:** 10.3390/molecules25184113

**Published:** 2020-09-09

**Authors:** Yanan Wang, Jinqing Jiang, Hanna Fotina, Haitang Zhang, Junjie Chen

**Affiliations:** 1 College of Animal Science and Veterinary Medicine, Henan Institute of Science and Technology, Xinxiang 453003, China; wyn564@126.com (Y.W.); histzht@126.com (H.Z.); 13462256164@vip.163.com (J.C.); 2 Faculty of Veterinary Medicine, Sumy National Agrarian University, 40021 Sumy, Ukraine

**Keywords:** total aflatoxin, antibody preparation, immunoassay, food safety

## Abstract

Aflatoxin (AF) contamination is a major concern in the food and feed industry because of its prevalence and toxicity. Improved aflatoxin detection methods are still needed. Immunoassays are an important method for total aflatoxin (TAF) analysis in food due to its technical advantages such as high specificity, sensitivity, and simplicity, but require high-quality antibodies. Here, we first review the three ways to prepare high-quality antibodies for TAF immunoassay, second, compare the advantages and disadvantages of antigen synthesis methods for B-group and G-group aflatoxins, and third, describe the status of novel genetic engineering antibodies. This review can provide new methods and ideas for the development of TAF immunoassays.

## 1. Introduction

Aflatoxins (AFs) are a group of toxic secondary metabolites containing similar molecular structures (difuran ring and oxyheteronaphthalidone). They are produced mainly by *Aspergillus flavu* and *Aspergillus parasiticus*, and other fungi such as *Aspergillus nomius, Aspergillus bombycis*, and *Aspergillus pseudotamari*, through the polyketone pathway. There are 20 kinds of AFs, and the AFs produced under natural conditions mainly include Aflatoxin B1 (AFB1), Aflatoxin B2 (AFB2), Aflatoxin G1 (AFG1), and Aflatoxin G2 (AFG2) [1,2]. AFs have toxic effects on human health such as acute poisoning, chronic poisoning, carcinogenicity, mutagenicity, malformation, neurotoxicity, and immunotoxicity. AFB1 is especially toxic, and most countries have clear limits on AFB1 residues in food [3,4]. In addition, AFB1, AFB2, AFG1, and AFG2 residues have synergistic negative effects on human health [5]. The detection of a single aflatoxin cannot meet the needs of the food industry. Maximum residue limits (MRL) and a corresponding detection method of total aflatoxin (TAF) in food are useful to regulate this material [6,7].

In view of this new TAF MRL standard and its role in the international food trade, many countries have studied TAF MRL standards and detection methods. To date, 91 countries have adopted TAF MRL standards and established corresponding detection methods. The International Codex Alimentarius Commission (CAC) stipulated that the TAF MRL for peanuts and their products should not exceed 15 μg/kg [8]. The U.S. food and drug administration (FDA) stipulated that the TAF MRL for all foods should not exceed 15 μg/kg [9]. The TAF MRL set by European Committee (EC) was no more than 15 μg/kg of peanuts and peanut products not directly used for human consumption or no more than 4 μg/kg of peanuts and peanut products and grains and products directly used for human consumption (no more than 10 μg/kg of agricultural corn and rice) [10]. Japan’s food safety law 0331-5 stipulated that all food TAF MRL should not exceed 10 μg/kg [11]. The current AF MRL standard of food and agricultural products in China is “GB 2761-2017 limit of fungal toxins in food”, which strictly stipulates the AFB1 MRL [12] and does not yet involve TAF MRL. However, the detection method of TAF MRL in feed is prescribed in “GB/T 30955-2014: immunoaffinity column purification and high-performance liquid chromatography” [13], and the detection method of TAF MRL in grain was prescribed in “LS/T 6128-2017: Determination of aflatoxin B1, B2, G1, and G2 in grain: ultra-high-performance liquid chromatography” [14].

TAF detection methods in food include physicochemical analysis and immunoassays. Physicochemical analysis methods are used in various countries. However, these techniques are expensive and lengthy and require complicated sample pretreatment procedures, expensive instruments, and skilled technicians. Thus, they are not suitable for high-throughput detection of many samples. Immunoassay methods based on the specific antigen–antibody reaction have been extensively used in the screening of TAF due to their high selectivity, strong sensitivity, rapid and simple sample screening, and portable operation [15]. The preparation of high-quality antibodies is a key technology to establish immunoassay methods. According to the current research progress, there are three ways to prepare high-quality antibodies for TAF immunoassay: The first is to prepare a single monoclonal antibody (mAb) of B-group AFs and G-group AFs with high sensitivity and strong specificity. These are then mixed with the universal antibody. The second is to prepare a single universal mAb capable of simultaneously recognizing AFB1, AFB2, AFG1, and AFG2 with high sensitivity and broad affinity. The third is the preparation of novel genetically engineered antibodies that could simultaneously identify AFB1, AFB2, AFG1, and AFG2 (recombinant antibody (rAb) including single chain fragment variable (scFv) and single-domain antibody (sdAb).

AFs are small molecules with no immunogenicity—they cannot directly induce the body to produce antibodies regardless of the pathway. Thus, it is necessary to combine them with macromolecular protein carriers to form artificial antigens with high immunogenicity. The proliferation and differentiation of B cells are indirectly induced by T-cell epitopes to prepare high-quality antibodies [16]. In this paper, research progress on antibody preparation in TAF immunoassay was reviewed in order to provide a reference for the development of the TAF immunoassays.

## 2. Preparation of the Mixed Universal mAb for TAF Immunoassay

### 2.1. Preparation of Sensitive and Specific mAb for B-Group AFs

**Figure 1:**
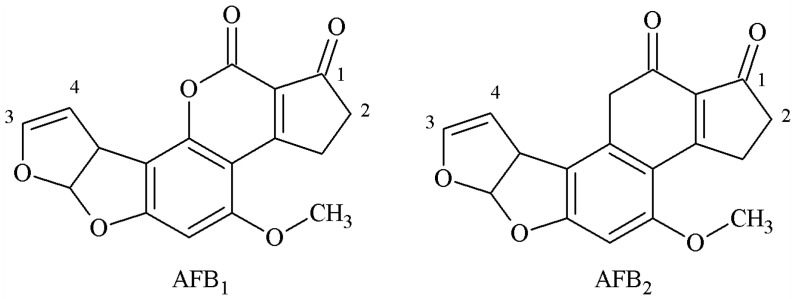
Molecular structure of AFB1 and AFB2.

B-group AFs are polycyclic unsaturated bisfuranolactone compounds containing a bisfuran ring structure related to basic toxicity and a coumarin structure related to carcinogenicity (Figure 1). According to the active groups and active sites on the molecular structure of B-group AFs, the synthesis methods of artificial antigen mainly included the oxime active ester (OAE), methylation of ammonia (MOA), mixed anhydride (MA), semi-acetal (SA), epoxide (EP), and enol ether derivative (EED) methods.

#### 2.1.1. OAE Method

**Figure 2:**
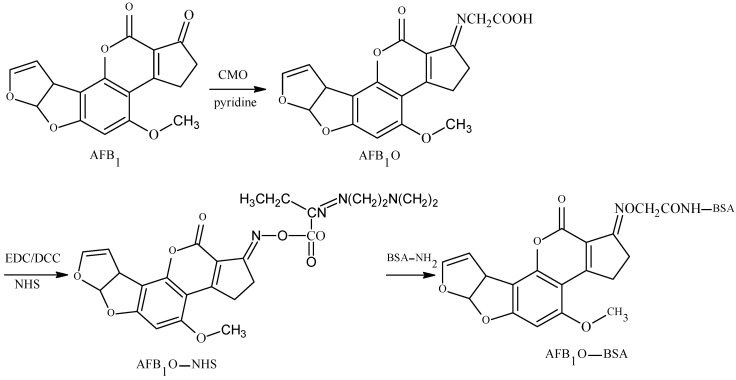
Synthesis of the AFB1-BSA antigen by the oxime active ester (OAE) method.

The 1-position carbonyl group of AFB1 was selected as the active site, and the carboxyl group was introduced by oximation with carboxymethoxylamine (CMO). The antigen was synthesized with a monoamide bond as the spacer arm. Chu et al. established the OAE method in 1977 [17]: This is the main method for the preparation of an AFB1-specific antibody and a TAF broad-spectrum antibody. Devi et al. prepared a high-specificity 10D5-1A11 mAb with a 50% inhibitory concentration (IC50) of 0.006 μg/kg and a limit of detection (LOD) of 0.001 μg/kg. The linear range was 0.001–1.0 μg/kg, and its cross reactivity (CR) with AFB2, AFG1, and AFG2 was 2%, 12%, and less than 1%, respectively [18]. Mohammad et al. prepared broad-spectrum A218 mAb—its IC50 was 0.006 μg/kg and its CR values with AFB2, AFG1, and AFG2 were 95%, 100%, and 100%, respectively [19]. The route of synthesis of the AflatoxinB1-Bovine serum albumin (AFB1-BSA) antigen by the OAE method is shown in Figure 2.

#### 2.1.2. MOA Method

**Figure 3:**
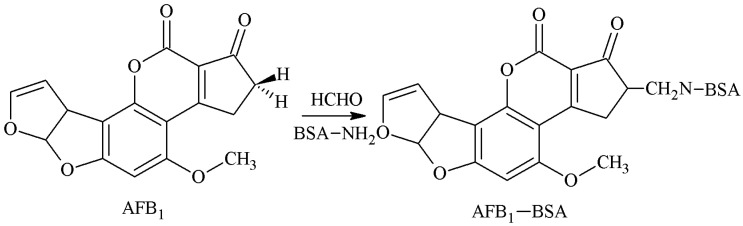
Synthesis of the AFB1-BSA antigen by the methylation of ammonia (MOA) method.

The 2-position α-active hydrogen was selected as the active site, and the AFB1-BSA antigen was synthesized by aminomethylation reaction with Mannich base as a spacer arm. The method was established by Wei et al. [20] and further improved by Fuentes et al. [21]. Urusov et al. [22] prepared an AFB1 polyclonal antibody (pAb) with an IC50 of 1.6 ng/mL and a CR of 82.6% of AFB2, but its specificity required further improvement. The route of synthesis of the AFB1-BSA antigen by the MOA method is shown in Figure 3.

#### 2.1.3. MA Method

**Figure 4:**
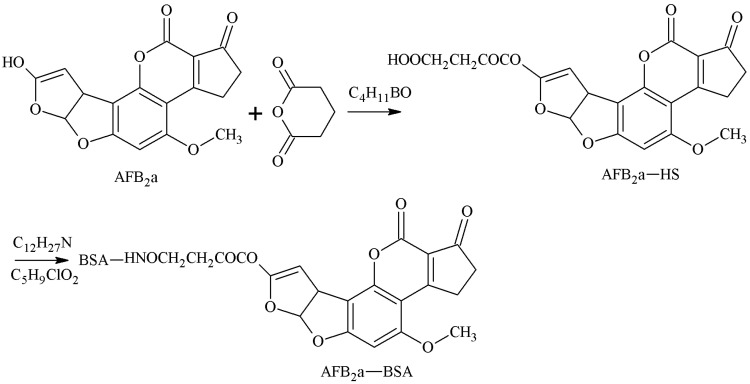
Synthesis of the AFB1-BSA antigen by the mixed anhydride (MA) method.

AFB1 was converted to AFB2a via an acid. The 3-position of the hydroxyl group of AFB2a was selected as the active site to react with anhydride to form a half-ester compound AFB2a-HS, and the AFB1-BSA antigen was synthesized with a monoamide bond as the spacer arm under the action of tri-n-butylamine and iso-butyl chloride. Lau et al. [23] established this method in 1980. Gaur et al. [24] later prepared AFB1 pAb, which could identify AFB1 100%. The CR of AFB2 reached more than 70%, but its sensitivity required further improvement. The route of synthesis of the AFB1-BSA antigen by the MA method is shown in Figure 4.

#### 2.1.4. SA Method

**Figure 5:**
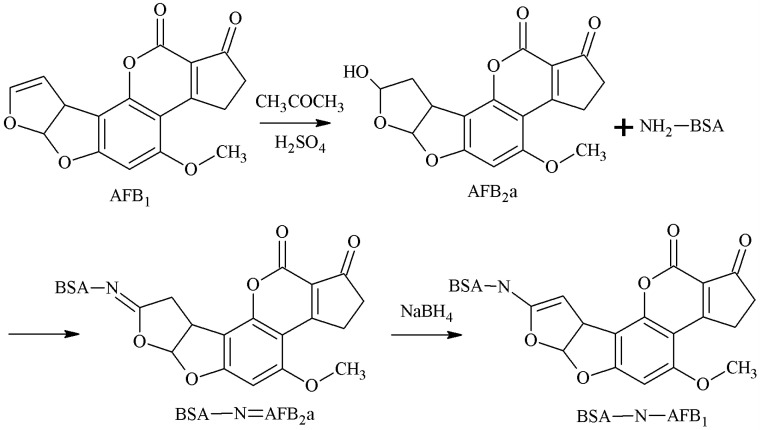
Synthesis of the AFB1-BSA antigen by the semi-acetal (SA) method.

AFB2a is the semi-acetal form of AFB1. The 3-position aldehyde group of AFB2a was selected as the active site, and the aldehyde group formed an unstable Schiff-base with the amino group of the carrier protein. Under the action of NaBH4, the AFB1-BSA antigen was synthesized via a monoamide bond as the spacer arm. Ashoor et al. [25] established this method in 1975. Xiao et al. [26] prepared a 3A12 mAb with this method, which had an IC50 of 6.1 μg/kg for AFB1, and CR values of 7.8%, 22.2%, and 0.6% for AFB2, AFG1, and AFG2 respectively, but its sensitivity needed to be improved. The route of synthesis of the AFB1-BSA antigen by the SA method is shown in Figure 5.

#### 2.1.5. EP Method

**Figure 6:**
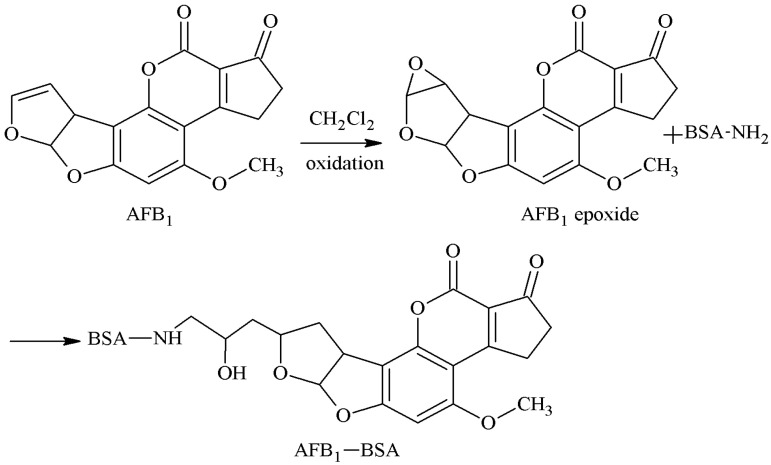
Synthesis of the AFB1-BSA antigen by the epoxide (EP) method.

The 3-4-position double-furan ring of AFB1 was selected as the active site, and the AFB1 epoxide was formed by oxidation. A hydroxyl group was introduced, the carboxyl group was introduced in the reaction with an anhydride, and the AFB1-BSA antigen was synthesized via a monoamide bond as the spacer arm. Martin et al. [27] established this method in 1977. Groopman et al. [28] later prepared AFB1 mAb, which could nicely identify AFB1 and AFB2. However, this method is rarely used now due to the strict reaction conditions, complicated reaction process, and low product yield. The route of synthesis of the AFB1-BSA antigen by the EP method is shown in Figure 6.

#### 2.1.6. EED Method

**Figure 7:**
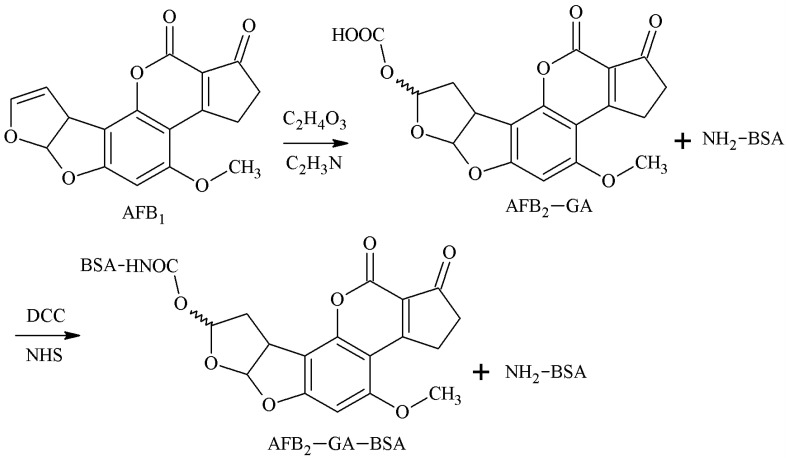
Synthesis of the AFB1-BSA antigen by the enol ether derivative (EED) method.

The 3-4-position double-furan ring of AFB1 was selected as the active site, and AFB1-glycolic acid was obtained via reaction with glycolic acid. The carboxyl group of the AFB1-glycolic acid was combined with a carrier protein to synthesize the antigen. Cervino et al. [29] established this method in 2007 and prepared AFB1 pAb and AFB1 mAb that 100% identified AFB1 and AFB2, but the sensitivity was poor. The route of synthesis of AFB1-BSA antigen by the EED method is shown in Figure 7.

These prior authors concluded that the OAE method had the best effect. The OAE method had simple operation steps, mild reaction conditions, and high product yield. The resulting antibodies had high efficiency, high sensitivity and specificity, and broad spectrum. The MA and SA methods were easy to perform. The antibody specificity was better, but the antibody sensitivity and broad spectrum need further improvement. The antibodies prepared by MOA, EP, and EED methods had many defects and were rarely used. The comparison of antigen synthesis methods of B-group aflatoxins are shown in Table 1.

### 2.2. Preparation of Sensitive and Specific mAb for G-Group AFs

**Figure 8:**
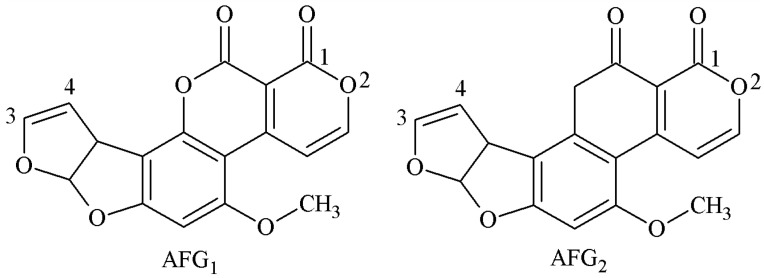
Molecular structure of AFG1 and AFG2.

The structure of G-group AFs is similar to that of B-group AFs—both of these contained a difuran ring and oxyheteronaphthalidone, except that the B-group AFs are connected to a pentanone, while the G-group AFs are connected to hexanolactone (Figure 8). The antigen synthesis of B-group AFs could be carried out through 1 and 2 sites, but the 1 and 2 sites of G-group AFs are very stable and difficult to achieve coupling with carrier proteins. The antigen synthesis of G-group AFs is mainly performed via 3 and 4 sites, and the synthesis methods include the SA method, EED method, and EP method.

#### 2.2.1. SA Method

**Figure 9:**
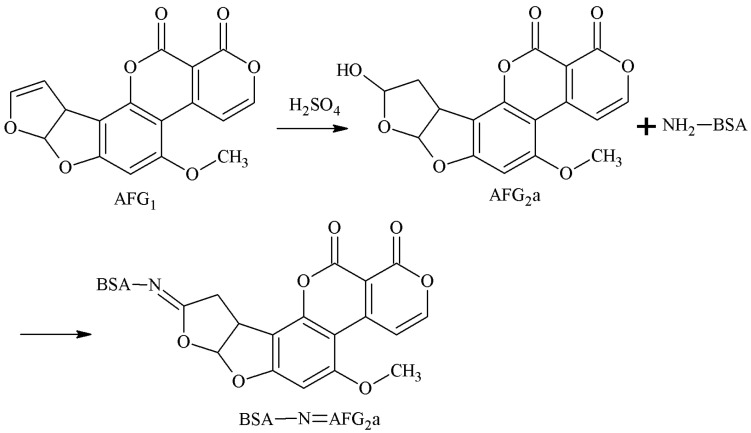
Synthesis of the AFG2a-BSA antigen via the SA method.

AFG1 was converted to AFG2a under acidic conditions, and the 3-4-position aldehyde group of AFB2a was selected as the active site. The aldehyde group of AFG2a formed an unstable Schiff-base with the amino group of the carrier protein. In the reduction of NaBH4, the AFB2a-BSA antigen was synthesized with a monoamide bond as the spacer arm. Chu et al. [30] established this method in 1985. Peiwu Li et al. [31] prepared AFG1 mAb with this method, and the IC50 of AFG1 and AFG2 was 17.18 and 19.75 μg/kg, respectively. The route of synthesis of the AFB2a-BSA antigen via the SA method is shown in Figure 9.

#### 2.2.2. EP Method

**Figure 10:**
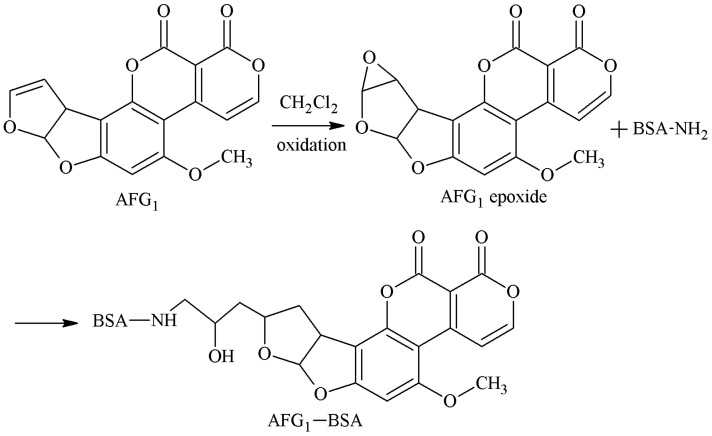
Synthesis of the AFG1-BSA antigen by the EP method.

The 3-4-position double-furan ring of AFG1 was selected as the active site. Under the oxidative action, the 3-4-position double-furan ring formed an epoxide that reacted with the amino groups of the carrier proteins to form secondary amines, and the antigen AFG1-BSA was synthesized via a monoamide bond as the spacer arm. Martin et al. [27] established this method in 1977. Zhang et al. [32] screened three hybridoma lines by this method, and the best was 1C8. The IC50 of AFG1 mAb secreted by 1C8 to AFG1 and AFG2 was 13.92 and 23.61 μg/kg, respectively. There was no CR for AFB1 and AFB2. The route of synthesis of the AFB2a-BSA antigen by the SA method is shown in Figure 10.

#### 2.2.3. EED Method

**Figure 11:**
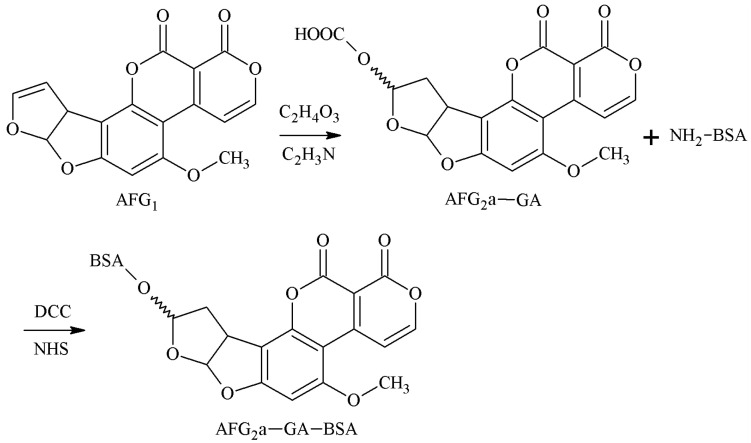
Synthesis of the AFG1-BSA antigen via the EED method.

The 3-4-position double-furan ring of AFG1 was selected as the active site, and the AFG1 enol ether derivatives (AFG1-GA) were obtained by adding glycolic acid junction arm, and the AFG1-GA carboxyl group was used to synthesize the antigen AFG1-BSA with BSA amino groups. Lyer et al. [33] established this method in 1993 and prepared AFG1 pAb and AFG1 mAb. These antibodies could identify AFG1 and AFG2 100% and had no CR with AFB1 and AFB2. However, the sensitivity of the antibodies was poor. The route of synthesis of the AFB2a-BSA antigen by the SA method is shown in Figure 11.

In summary, the SA method had the best effect of the three methods of artificial antigen synthesis of G-group AFs. This method was simple to operate with mild reaction conditions and high product yield, and the resulting antibodies had a high titer, high sensitivity, and good specificity. The EP method and EED method had poor sensitivity and were rarely used.

## 3. Preparation of a Single Universal Antibody for TAF Immunoassay

Current results show that AFB1 was selected as the starting material for the reaction—a single universal TAF mAb was prepared by the OAE method. Chu et al. established this method in 1977, and AFB1O yields were 73%–83% [17]. Kolosova et al. [34] and Cervino et al. [35] improved the method, and the yield of AFB1O was increased to more than 90%. Although the pAb against AFB1 was obtained, its CR with AFB2, AFG1, and AFG2 was lower and could not be used to establish the immunoassay method for TAF.

Zhang et al. [36] used this method to screen five hybridoma lines of 1D3, 4F12, 1C11, 10G4, and 4F3, all of which could be used for the detection of TAF. The 1C11 had the best effect, and the IC50 values of 1C11 mAb for AFB1, AFB2, AFG1, and AFG2 were 1.2, 1.3, 2.2, and 18.0 pg/mg. The CR values of 1C11 mAb for AFB1, AFB2, AFG1, and AFG2 were 100%, 92.3%, 54.5%, and 6.7%, respectively. Kim et al. [37] used this method to screen the 8H10 hybridoma line with IC50 value for AFB1, AFB2, AFG1, and AFG2 of 4.36, 7.22, 6.61, and 29.41 μg/kg. The CR values of the 8H10 mAb for AFB1, AFB2, AFG1, and AFG2 were 100%, 60.47%, 65.97%, and 14.83%, respectively. Li et al. [38] reported that the dose of immunogen had a great contribution to the specificity and broad spectrum of AFs mAb, and the low dose of immunogen helped to obtain a specific narrow-spectrum mAb. The high dose of immunogen helped to obtain a specific broad-spectrum mAb. Immunogen AFB1-BSA was prepared via the OAE method, and each Balb/c mouse was immunized with 150 μg immunogen. The specific and broad-spectrum 4F3 mAb was obtained, and its IC50 for AFB1 was 0.29 μg/kg. The CR values for AFB1, AFB2, AFG1, and AFG2 were 100%, 171%, 200%, and 57%, respectively.

Immunoglobulin(Ig) antibodies include immunoglobulin G (IgG), immunoglobulin A (IgA), immunoglobulin M (IgM), immunoglobulin E (IgE), and immunoglobulin D (IgD). IgG antibodies are the most common mAb and are core reagents for immunoassays due to their high specificity, affinity, and binding capacity. IgM, IgE, and IgD antibodies generally do not have a mature affinity and are rarely used in immunoassays for small molecule haptens. IgA antibodies are polyvalent and have high affinity due to their four binding sites [39]. Ertekin et al. [40] reported that immunogen AFB1-HTF-BSA was prepared by the MOA method with AFB1 as the starting material and human apo transferrin (HTF) as the carrier protein. IgA D12E2 mAb was prepared by immunizing Balb/c mice and cloning hybritoma, which could well identify AFB1, AFB2, AFG1, and AFG2. The IgA D12E2 mAb established the TAF immunoaffifinity column (IAC) and enzyme-linked immunosorbent assay (ELISA) method. The total binding capacity of IAC was 111, 70, 114, and 73 ng for AFB1, AFB2, AFG1, and AFG2, respectively. The detection limit of ELISA for TAF was 2 µg/kg, and the detection range was 2–50 µg/L.

## 4. Preparation of Novel Genetically Engineered Antibodies for TAF Immunoassays

### 4.1. Preparation of scFv

The scFv is a recombinant protein composed of a variable region of heavy chain (VH) and variable region of light chain (VL) of the antibody through a short peptide of 15–25 amino acids. Bird et al. and Huston et al. developed the first successful scFv in 1988 [41,42]. The scFv have a small molecular weight, strong penetration, and high affinity. They have been widely used in tumor therapy, infectious disease prevention and treatment, food safety residue detection, and other fields [43].

**Figure 12:**
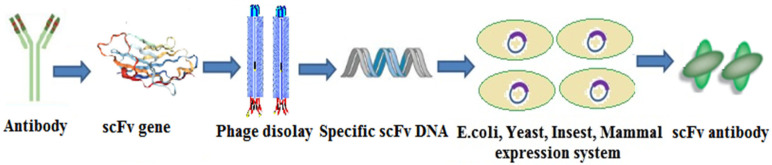
The scFv preparation process.

The preparation of scFv is divided into three steps. In the first step, the VH and VL genes were amplified by the PCR technique and then connected by a Linker. Second, the connected genes were introduced into the display system for display and screening. Commonly used scFv display systems included phage display, ribosome display, and yeast surface display. Third, the screened connected genes were transferred into the expression system for mass production. Common scFv expression systems include prokaryotic expression systems, eukaryotic expression systems, and plant expression systems [44]. The scFv preparation process is shown in Figure 12.

Good progress has been made in the preparation and application of AFs scFv. Moghaddam et al. first used phage display technology to successfully prepare AFs scFv, which could specifically bind to AFB1 with an affinity of 6 × 10^−9^ moL/L [45]. The TAF universal antibody of AFB1 8F6 mAb was prepared by Li et al., and its sensitivity to AFB1, AFB2, AFG1, and AFG2 reached 1.7, 1.63, 1.69, and 3.60 μg/kg, respectively. The genes were constructed by PCR linking the VH and VL genes of AFB1 8F6 mAb, and the scFv genes were cloned onto phage expression vector pCANTAB5E, so that the scFv genes were expressed in TG1 of *Escherichia coli* in phage display form. The expression product was used to establish an ELISA detection method for TAF immunoassay by scFv [46]. Li et al. [47] constructed an AFB1 scFv library with a storage capacity of 3.5 × 10^5^ Colony-Forming Units (CFU) by extracting the total RNA from more than 20 AFB1 mAb hybridioma lines, which laid a good technical and material foundation for establishing the TAF immunoassay with scFv.

### 4.2. Preparation of Single-Domain Antibody

In 1989, Muyldermans found that half of the antibodies in camel blood naturally had no light chains (LC) and heavy chain constant regions (CH). Through in-depth research, in 1993, Muyldermans and his mentor professor Hamers reported the heavy chain antibodies (HCAbs) in Nature [48]. Researchers subsequently identified a new Ig antigen receptor (IgNAR) similar to HCAbs in chondrocytes [49]. HCAbs-specific antigen binding is a variable region of a single heavy chain, called the variable domain of the heavy chain of heavy-chain antibody (VHH). The single-domain antibody (sdAb) is the smallest antigen-binding unit of the antibody and consists of either only one variable domain or one engineered constant domain that solely facilitates target binding [50]. The sdAb is usually composed of only 110–130 amino acids with an oval shape of 2.5 nm in diameter, 4 nm in length, and a relative molecular weight of only 15 kDa. This is also called a nanobody (Nb) [51,52]. The sdAb has a small molecular weight, easy expression, strong penetration, high specificity, high affinity, good solubility, good stability, low immunogenicity, and simple humanized technology compared with traditional antibodies. It has been widely used in basic biological research, medical diagnosis, drug development, food safety detection, and other fields [53,54].

**Figure 13:**
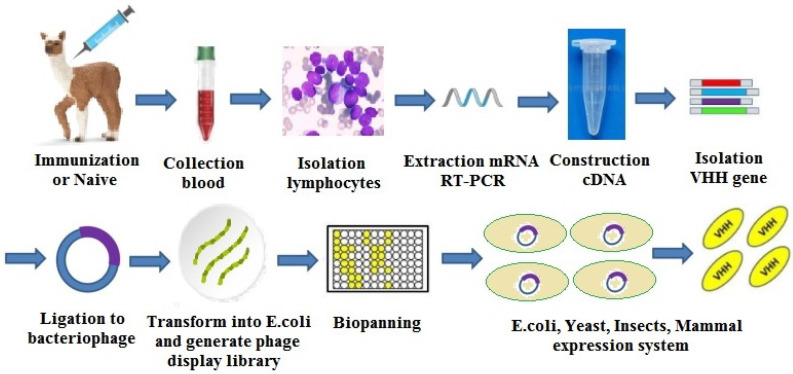
The single-domain antibody (sdAb) preparation process.

The sdAb are usually prepared via phage display library techniques. According to the source of antibody gene, a phage sdAb library could be divided into three categories: natural library, immune library, and synthetic library. The establishment and screening of a phage sdAb library include three main steps: acquisition of the antibody gene fragment, panning of the target gene, and expression/purification of the antibody. The sdAb preparation process is shown in Figure 13 [54].

Recently, the preparation of mycotoxin sdAb and the establishment of immunoassay methods based on the sdAb have been reported, such as deoxynivalenol (DON) [55], Zearalenone (ZEN) [56], Ochratoxin A (OTA) [57], and fumonisin B1 (FB1) [58]. Liu et al. [59] reported the preparation of TAF sdAb and the establishment of immunoassay methods based on the TAF sdAb. More specifically, this work reported the preparation of positive clones targeting TAF sdAb Nb5 and Nb13 based on the constructed phage display sdAb natural library using AFB1-BSA as a target molecule, AFB1 as the competitor, and solid-phase panning techniques and phage-ELISA to identify positive clones. However, the sensitivity of Nb5 and Nb13 required further improvement.

Wang et al. [60] reported that AFB1-BSA was used to immunize alpaca to construct a phage display sdAb immune library, and three TAF sdAb were screened. Of these, the IC50 of Nb25 against AFB1 was 0.16 ng/mL, and the CR value for AFB2, AFG1, and AFG2 was 90.4%, 54.4%, and 37.7%, respectively. A TAF ELISA method was established by Nb25, and it was successfully used to detect TAF in peanut, corn, and rice. The conventional TAF of ELISA was compared with the TAF ELISA based on Nb25, and the results showed a good correlation (r^2^ = 0.89). Ren et al. [61] used the Nb25 to establish a method for quantitative detection of TAF in grain by phage display-mediated immuno-polymerase chain reaction (PD-IPCR). The IC50 value of AFB1, AFB2, AFG1, and AFG2 was 0.43, 0.45, 1.22, and 3.41 μg/kg, respectively. The detection range of TAF was 0.09–2.7 μg/kg for wheat samples, 0.1–5.7 μg/kg for corn samples, 0.14–6.1 μg/kg for rice samples, and 0.04–6.9 μg/kg for feed samples.

## 5. Conclusions and Prospects

Traditional mAb still plays a key role in TAF immunoassays. The preparation of TAF mAbs with strong specificity, wide recognition spectrum, and high affinity is critical. For the synthesis of AF antigens, both empirical design and predictive design are mostly performed via trial-and-error assays, with disadvantages of relatively high blindness and contingency [16]. The theory of hapten molecular design and antigen synthesis includes the complexity of hapten spatial structure, active sites, the introduction of active groups, and length of entering spacer arm [62,63]. These continue to be improved with molecular immunology, computational science, quantum chemistry, molecular dynamics, and molecular simulations. Kim et al. prepared TAF mAb with strong specificity and a broad recognition spectrum through computer-aided technology and realized TAF immunoassay via ELISA [37].

In recent years, some achievements had been made in the theoretical research and technological progress of genetically engineered antibodies, but their applications in the field of food safety detection remain in the experimental research stage. Such research mainly focuses on the preparation and application of scFv and sdAb [64].

There are four problems with TAF scFv and TAF sdAb. First, there are deviations in the PCR amplification and expression of antibody genes that affect the specificity. Second, the affinity of TAF scFv and TAF sdAb is low, which affects the sensitivity. Third, the expression efficiency of TAF scFv and TAF sdAb should be further improved. Fourth, the stability of TAF scFv and TAF sdAb is poor, which affects the accuracy [65,66]. Site-directed mutagenesis, error-PCR, chain shuffling, DNA shuffling, and other technologies can improve the performance of TAF scFv and TAF sdAb and play an important role in TAF immunoassays.

In addition, antibody engineering can lead to better genetically engineered antibodies such as recombinant antibody fragment of antigen binding (Fab) [66] and multivalent antibodies [67]. Although the preparation and application of these antibodies is theoretical with technical testing, this area is a key new direction of antibody research. In the near future, these new antibodies will replace the original traditional antibodies and play a more active role in the field of rapid detection of food safety analysis.

## Figures and Tables

**Table 1:** Comparison of antigen synthesis methods of B-group aflatoxins.

Synthetic Methods	Active Sites	Reaction Principle	Advantages	Disadvantages
OAE	1	The 2-carbonyl group of AFB1 was selected as the active site, the active group carboxyl was introduced by oximation, and the monoamide bond was used as the spacer arm to synthesize the antigen.	The antibodies prepared had good specificity and broad spectrum, which was the main way to realize TAF immunoassay. The reaction conditions were mild and the product yield was high.	The high price of test material results in high test cost.
MOA	2	The 2-active hydrogen of AFB1 was selected as the active site, and the antigen was synthesized with Mannich base as the spacer arm through ammonia-methylation reaction.	The experimental operation was simple, the reaction condition was mild, and the product yield was low.	The antibodies prepared had poor specificity.
MA	3	The 3-hydroxyl group of AFB2a was selected as the active site and reacted with anhydride to form the hemiester compound AFB2A-HS. Monoamide bond was used as the spacer arm to synthesize the antigen under the action of tri-butylamine and isobutyl chloroformate.	The experimental operation was simple, the reaction conditions were mild, and the product yield was high.	The antibodies prepared had poor sensitivity.
SA	3	The 3-position aldehyde group of AFB2a was selected as the active site, and the aldehyde group of AFB2a and the amino group of the carrier protein generated unstable Schiff-base. Under the action of NaBH4, monoamide bond was used as the spacer arm to synthesize the antigen.	The antibodies prepared had good specificity. The experimental operation was simple.	The experiment operation was complicated and the product yield was low. The antibodies prepared had poor sensitivity.
EP	3, 4	The 3 and 4-position furan ring of AFB1 was selected as the active site, AFB1 epoxides were formed through oxidation, hydroxyl groups were introduced, carboxyl groups were introduced in reaction with anhydride, and monoamide bond was used as the spacer arm to synthesize antigen.	The antibodies prepared had good specificity and broad spectrum.	The reaction condition was strict, the experiment operation was complicated, and the product yield was low. The antibodies prepared had poor sensitivity.
EED	3, 4	The 3 and 4-position furan ring of AFB1 was selected as the active site, and afB1-Glycolic Acid was obtained by reacting with Glycolic Acid. The carboxyl group of AFB1-Glycolic Acid was conjured with carrier protein to form an antigen.	The experiment operation was simple and the product yield was high.	The specificity and sensitivity of the prepared antibodies were poor.

OAE: oxime active ester, MOA: methylation of ammonia, MA: mixed anhydride, SA: semi-acetal, EP: epoxide, EED: enol ether derivative.
